# Angiogenic ability of human endothelial cells was decreased following senescence induction with hydrogen peroxide: possible role of vegfr-2/akt-1 signaling pathway

**DOI:** 10.1186/s12860-022-00435-4

**Published:** 2022-07-25

**Authors:** Nesa Janamo Berenjabad, Vahid Nejati, Jafar Rezaie

**Affiliations:** 1 Department of Biology, Urmia University, Urmia, Iran; 2 Solid Tumor Research Center, Cellular and Molecular Medicine Institute, Urmia University of Medical Sciences, Shafa St, Ershad Blvd, P.O. BoX: 1138, Urmia, Postal Code: 57147 Iran

**Keywords:** Angiogenesis, HUVECs, H_2_O_2_, Age-related diseases, Senescence

## Abstract

**Background:**

Many attempts are used to discover mechanisms driving impaired angiogenesis in age-related diseases. Angiogenesis is highly regulated by different signaling pathways. Here, we investigated the angiogenesis potential of human endothelial cells (ECs) upon exposure to hydrogen peroxide (H_2_O_2_), a cellular senescent factor.

**Results:**

Data showed that the wound healing rate of HUVECs decreased upon incubation with H_2_O_2_ (*P* < 0.05). LOX activity and NO production were decreased in H_2_O_2_ treated cells (*P* < 0.05). Expression of miR-126 and VEGFR-2 up-regulated, while expression of miR-373 and HSP-70 up = regulated in H_2_O_2_ -induced cells (*P* < 0.05). In addition, we found that protein levels of p-Akt-1, VCAM-1, MMP-9, and IL-6 decreased in treated cells (*P* < 0.05).

**Conclusions:**

Our data showed that H_2_O_2_ reduced the angiogenic response of HUVECs in vitro, which may be due to impairment of the VEGFR-2 signaling pathway.

**Supplementary Information:**

The online version contains supplementary material available at 10.1186/s12860-022-00435-4.

## Background

Angiogenesis, remodeling and formation of the new blood vessel, contributes to physiological processes like wound healing and embryonic development [1, 2]. Highly structured, angiogenesis is a multistep process, regulated by different cytokines, pro/anti-angiogenic factors, and angiogenic cells like endothelial cells (ECs) and mural cells [3, 4]. Angiogenesis responses are depended on the balance between antiangiogenic and proangiogenic factors present in the microenvironment [3–5]. Improper or dysfunctional angiogenesis facilitates pathologies processes including cardiovascular diseases (CVD), atherosclerosis, cancer, and diabetes [6, 7]. ECs line the inner layer of the cardiovascular system, providing a natural barrier between the rest of the tissues and the blood [8]. This monolayer is metabolically active and continuously faces various biochemical and biomechanical factors, responds properly, keeps the homeostasis and integrity of vascular function [8]. Impaired endothelium resulting from ECs dysfunction, a primary feature of vascular diseases, is associated with different diseases such as CVD [8, 9]. According to studies, aging is the main reason for endothelial dysfunction [10, 11], compromising tissue perfusion and worsening functional debility in older persons. Alterations caused by aging may impact ECs phenotype and signaling pathways, losing the structural and mechanical integrity of the circulatory system [10, 11]. The dynamic of angiogenesis is associated with vascular integrity and regression which is critical for the microvascular network maintenance in the heart and other organs. Age-related angiogenesis impairment and consequential impaired microvascular homeostasis promote the pathogenesis of diseases [12–14]. The mechanisms involved in age-related impaired angiogenesis are complex and probable to include increased nitrative and oxidative stress and changes in the well-preserved molecular pathways affecting mutual ageing processes [12, 13]. Impaired angiogenesis possibly results from declined nitric oxide bioavailability, metabolic dysregulation, apoptosis, extracellular matrix remodeling, impaired pericyte function, and alterations in cytokines and angiogenic-related factors [13, 15]. Several signaling such as ROS may inactivate nitric oxide and subsequent cause ECs and vasomotor dysfunction [13]. CVD is the leading cause of death and disability among the elderly population, with a burden on healthcare worldwide. Age-related death from CVD exponentially rises with age during the later years of life [12]. Several lines of evidence support the concept that aging causes phenotypic alterations that render the coronary circulation disposed to disease even in the absence of traditional risk factors including metabolic diseases, hypertension, and smoking [13, 16]. An understanding of the underlying mechanisms implicated in the age-related impairment of angiogenesis is vital for decreasing CVD-related mortality in aging people. The main purpose of this study is to develop an understanding of angiogenesis signaling under H_2_O_2_ exposure focus on VEGFR-2 downstream signaling. We induced aging using H_2_O_2_ and human umbilical vein endothelial cells (HUVECs) as ECs models for angiogenesis studding.

## Results

### H2O2 reduced the wound healing rate of HUVECs

**Fig. 1:**
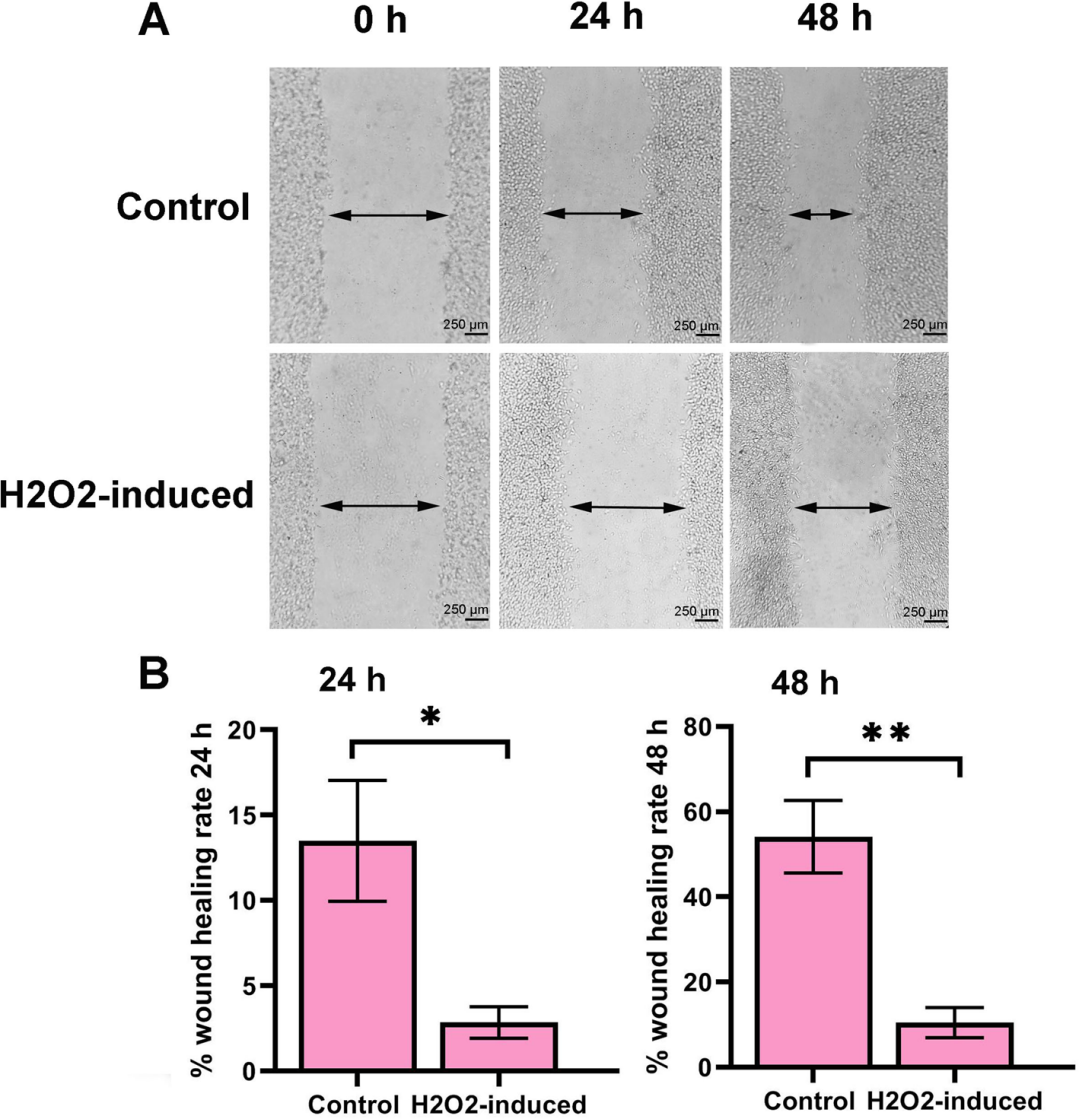
The wound healing rate of human endothelial cells (HUVECs) was measured via in vitro scratch assay throughout 24 h and 48 h (**A**). Result showed that wound healing rate of treated cells was decreased after 24 h and 48 h (**B**). Data are prepared as mean ± standard deviation. The means were compared using a T-test. *P < 0.05 and **P < 0.01. n = 3. Scale bar: 250 µm

To determine the effect of H_2_O_2_ on the wound healing rate of HUVECs, in vitro scratch assay was used. As shown by Fig. 1, we found that the wound healing rate of H_2_O_2_ -induced cells significantly decreased compared to control cells after 24 h of incubation with H_2_O_2_ (*P* < 0.05). Similarly, we also observed that the wound healing rate of cells decreased throughout 48 h (*P* < 0.01). Data showed that the H_2_O_2_ prevented the migration of cells to the scratched area.

### LOX activity of cells was decreased in treated cells

**Fig. 2:**
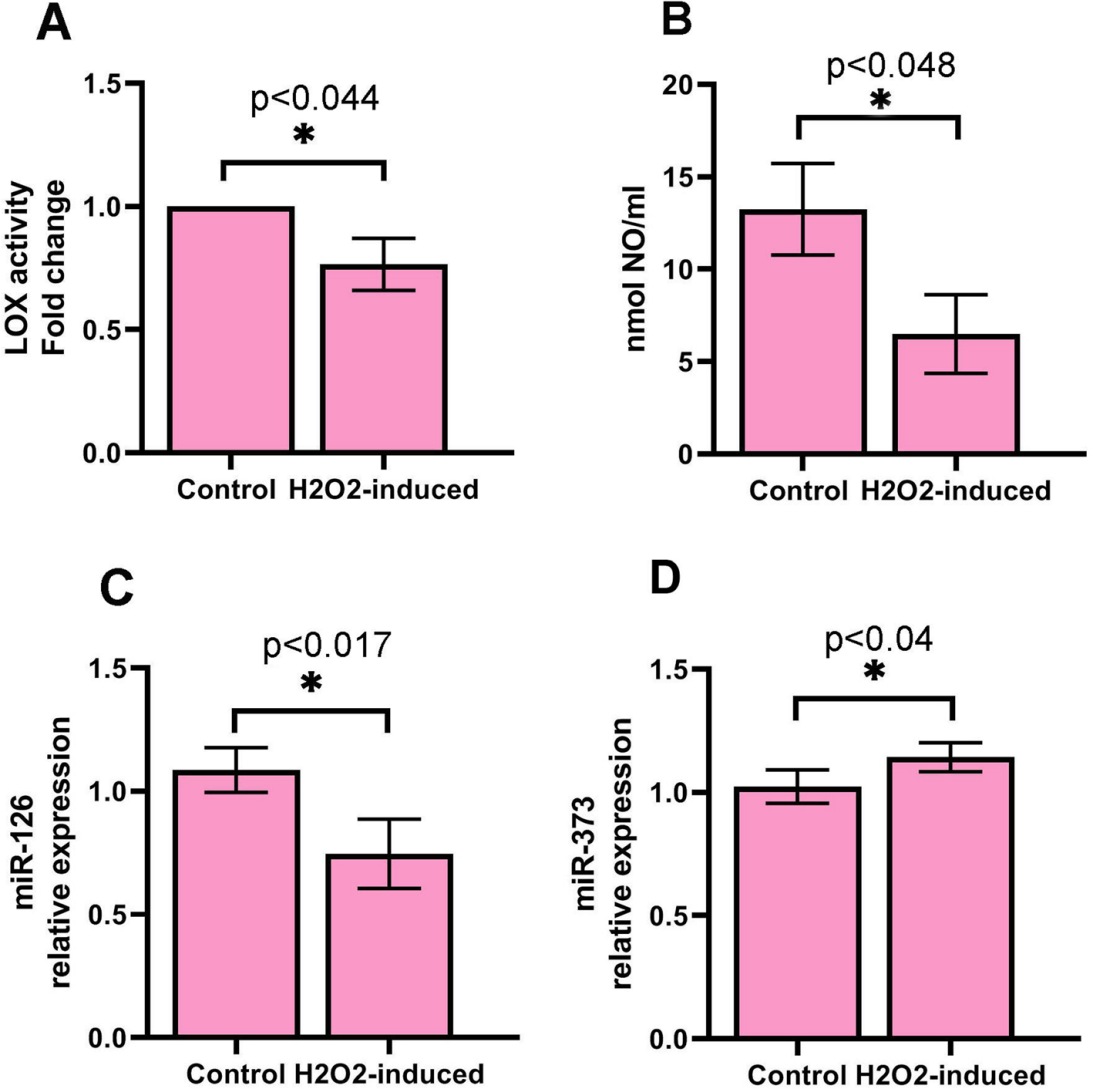
LOX activity and NO production of HUVECs were analyzed by colorimetric methods (**A** and **B**). Real-time PCR was used to monitor the expression of miR-126 and miR-373 in HUVECs (**C** and **D**). Data are prepared as mean ± standard deviation. The means were compared using a T-test. *P < 0.05 and **P < 0.01. *n* = 3

To monitor the level of LOX enzyme activity in cells, LOX activity was measured.Results showed that the activity of the LOX enzyme was inhibited in treated HUVECs (0.765 ± 0.106 fold change) compared to control cells (*P* < 0.05, Fig. 2A).

### NO production was decreased in treated cells

To measure the level of NO concentration in cells subjected to H_2_O_2_, we did a Griess assay. Results showed that NO concentration was significantly declined in treated HUVECs compared to control cells (*P* < 0.05; Fig. 2B).

### Expression of miR-126 and miR-373 was changed in treated cells

We also calculated the expression of pro-angiogenic miRs in treated HUVECs by q-PCR assay. As shown by Fig. 2, compared to control cells, we observed that level of miR-126 was decreased in treated cells (1.087 ± 0.09 vs. 0.746 ± 0.14; *P* < 0.05, Fig. 2C). In addition, the expression of miR-373 was down-regulated in treated cells compared to control cells (1.023 ± 0.069 vs. 1.143 ± 0.053; *P* < 0.05, Fig. 2D).

### Expression of angiogenic genes was altered in treated cells

**Fig. 3:**
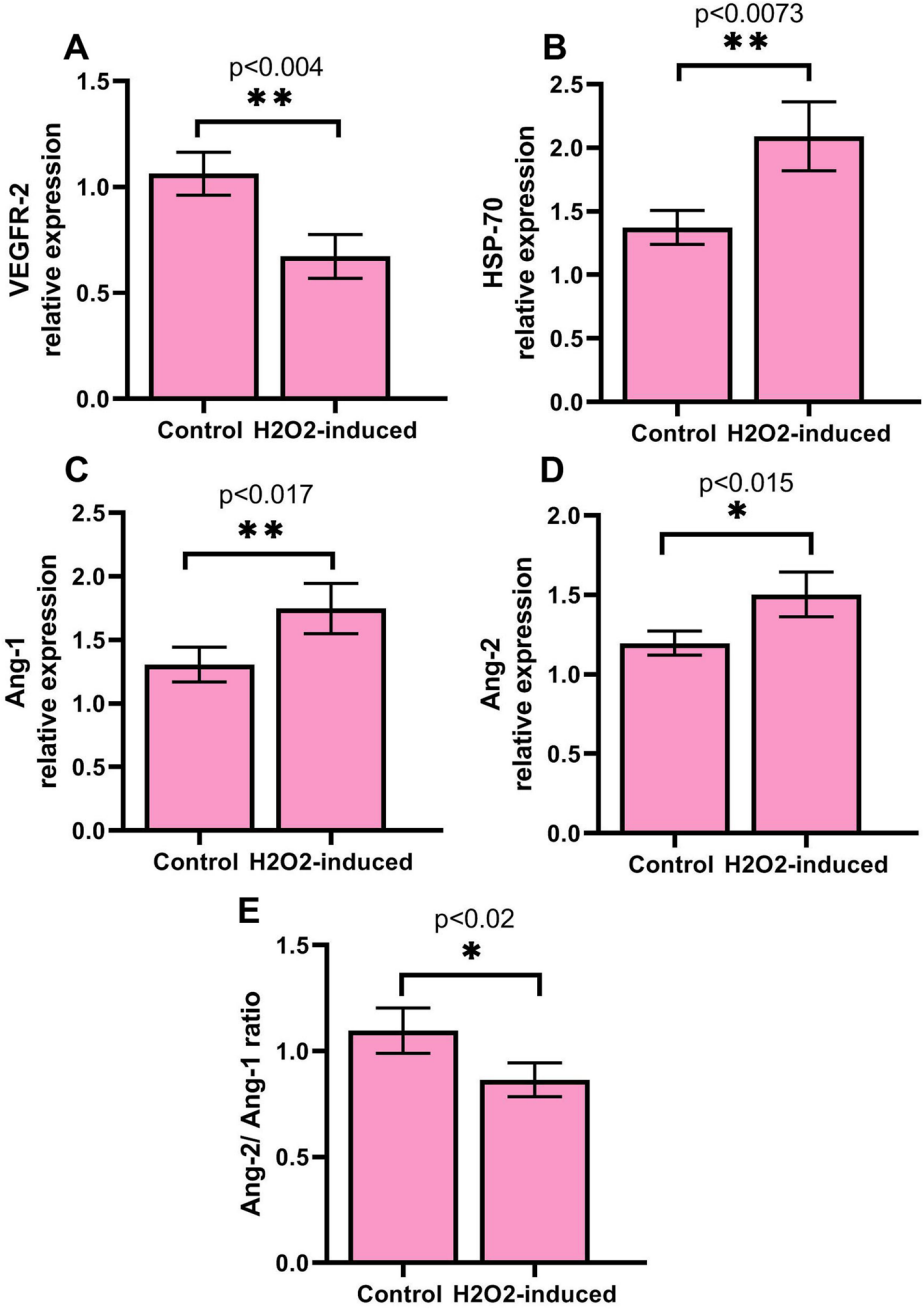
The relative expression of angiogenic genes including VEGFR-2 (**A**), HSP-70 (**B**), Ang-1 (**C**), Ang-2 (**D**), and Ang-2/Ang-1 ratio (**E**) were calculated by real time-PCR. The expression of genes was normalized against the GAPDH gene. Data are prepared as mean ± standard deviation. The means were compared using a T-test. *P < 0.05 and **P < 0.01. *n* = 3

To further assess the effect of H_2_O_2_ on genes involved in angiogenesis including VEGFR-2, Ang-1, Ang-2, and HSP-70, Q-PCR assay was used. Data revealed that expression of VEGFR-2 (1.066 ± 0.1 vs. 0.6727 ± 0.1;, *P* < 0.05) decreased, while expression of Ang-1 (1. 3 ± 0.13 vs. 1.747 ± 0.19;), Ang-2 (1.197 ± 0.076 vs. 1.503 ± 0.14), and HSP-70 (1.37 ± 0.13 vs. 2.01 ± 0.27) increased in treated HUVECs (*P* < 0.01; Fig. 3). Furthermore, we found that the Ang-2/Ang-1 ratio was decreased in treated HUVECs (1.097 ± 0.1 vs. 0.8647 ± 0.079) (*P* < 0.05; Fig. 3).

### H2O2 decreased protein levels of p-Akt-1, VCAM-1, MMP-9, and IL-6 in treated cells

**Fig. 4:**
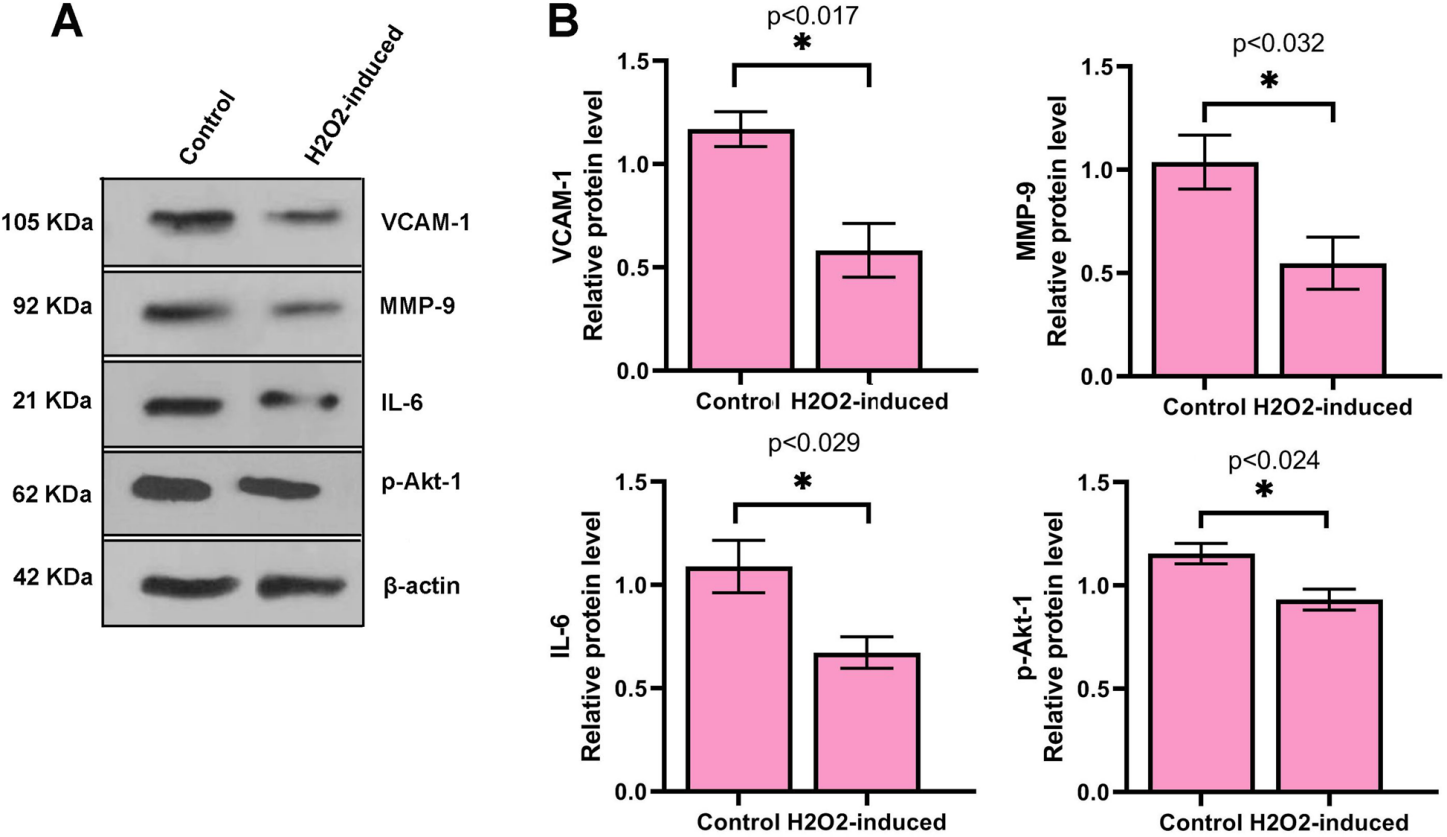
The relative VCAM-1, MMP-9, IL-6, and Akt-1 protein levels were quantified to β-actin using western blotting analysis (**A** and **B**). Data are prepared as mean ± standard deviation. The means were compared using a T-test. *P < 0.05 and **P < 0.01. *n* = 3

We performed a western blotting assay to measure protein levels of p-Akt-1, VCAM-1, MMP-9, and IL-6 in cells. The protein level of p-Akt-1 was decreased in treated cells (1.15 ± 0.049 vs. 0.93 ± 0.05; *P* < 0.05; Fig. 4). In addition, we observed a significant decrease in the protein level of VCAM-1 (1. 17 ± 0.091 vs. 0.58 ± 0.13) and MMP-9 (1. 046 ± 0.13 vs. 0.548 ± 0.12), and IL-6 (1. 075 ± 0.12 vs. 0.673 ± 0.076) compared to the control. These findings showed that expression of pro-angiogenic proteins decreased in cells incubated with H_2_O_2_.

## Discussion

Angiogenesis is a preserved process by which new capillaries develop from vascular bed and is essential for numerous biological events, comprising embryonic development, regeneration and wound healing. In pathological conditions, the multifarious synchronized activities of several pro-and anti-angiogenic factors regulate angiogenesis [17]. Despite growing interest in senescent-induced angiogenesis, new therapeutic attitudes and molecular mechanisms in H_2_O_2_ -induced angiogenesis have not been extensively scrutinized yet [18, 19]. Aging also changes pro-and anti-angiogenic mediators, causing dysregulated accessibility of these mediators in various tissues [20, 21]. Here, we explored the angiogenesis signaling in human ECs using H_2_O_2_, as an oxidative stress inducer promoting cellular senescence.

Our previous studies showed that H_2_O_2_ treatment induced cellular senescence in HUVECs [22, 23]. In this study, we show that, in response to H_2_O_2_ treatment wound healing rate of cells decreased, the feature is associated with the migration and proliferation ability of ECs. Similarly, Loo et al*.* by using a mice wound model, found that a high concentration of H_2_O_2_ delayed angiogenesis and wound closure [24]. This observation highlights the impairment of migration responses of ECs against H_2_O_2_, however, we further investigated the possible mechanisms. We measured the LOX activity of cells, an activity related to the mobility of cells, formation, and remodeling of extracellular matrix (ECM). We found that LOX activity of HUVECs was decreased upon treatment with H_2_O_2_, which may correlate to the wound healing ability of cells [25]. Besides these findings on H_2_O_2_ -induced cells, we found that expression of VEGFR-2, a primary mediator of angiogenesis, significantly reduced. Kusmartsev et al*.* reported that the treatment of myeloid cells with H_2_O_2_ caused an increase in expression of VEGFR1 [26]. H_2_O_2_ treatment induced lipid peroxidation product 4-hydroxy-2(E)-nonenal, or an inhibitor of thioredoxin reductase all resulted in up-regulation of VEGFR1. They also found that myeloid cells acquire immunosuppressive properties and could inhibit T cell proliferation. Findings indicated that tumor-induced oxidative stress may induce both VEGFR1 up-regulation and immunosuppressive function in myeloid cells [26]. VEGFR-2/VEGF signaling is critical for ECs migration and angiogenesis [27]. These may reflect the inhibitory effect of H_2_O_2_ on HUVECs mobility and migration. We further investigated the expression of Ang-1 and Ang-2 genes involved in the remodeling of the vasculature, regulating angiogenesis. Ang-1 participates in stabilizing vascular bed, whereas Ang-2 is an Ang-1 antagonist that disrupts the networks between the ECs and perivascular cells therefore destabilizes vascular integrity. Furthermore, a decrease in VEGFR-2 may participate in down-regulating Ang-2 compared to the Ang-1 gene [28]. In this regard, a decreased value in the Ang-2/Ang-1 ratio was observed, confirming a decrease in angiogenesis of treated cells. Seemingly, the increased levels of Ang-2 and Ang-1, as well as decreased Ang-2/Ang-1 ratio and VEGFR-2, participate in the low ability of HUVECs in inducing angiogenesis [29, 30]. Similarly, it was demonstrated that expression of Ang-2 was increased in human myoblasts by H_2_O_2_-treatment [31]. Increased level of Ang-2 significantly promoted myoblast survival and differentiation, but had no effect on cell proliferation and migration. Authors reported that Ang-2 increased cell survival through activation of the ERK1/2 and PI-3 kinase/Akt pathways [31]. Although, we did not measure this signaling, it is possible that Akt signaling interplay with the Ang-2 for ECs activity [31, 32]. Furthermore, we also demonstrated that the expression of HSP-70 was increased. Under stress conditions, the expression of HSP-70 is increased [33]. In our recent study (data unpublished), we found that oxidative stress induced by H_2_O_2_ could initiate autophagy flux, which may relate to an increase in HSP70 level in treated cells. However, HSP-70 has been reported to regulate angiogenesis [34]. Simard et al*.* reported that HSP-70 can promote migration of lymphocytes [35], although Kasioumi and co-workers observed that suppression of HSP-70 decreases cells migration [36]. It seems that further examination is needed to explain the possible interactions regarding the HSP-70 pathway.

To explore further angiogenesis responses, in agreement with Kumar et al*.* study, we found that NO concentration decreased [37]. Coincided with this observation, we found a decrease in VCAM-1 protein level. These findings and results of VEGFR-2 motivated us to scrutinize possible underlying mechanisms, therefore, we measured p-Akt-1 protein, an upstream regulator protein in VEGFR-2/Akt/eNOS/VCAM-1 axis [38]. Our findings showed that the p- Akt-1 protein level was significantly decreased in treated cells, suggesting an impaired angiogenesis [39, 40]. Akt protein can regulate different signaling pathways and play key roles in multiple cellular processes such as cell death, glucose metabolism, transcription, proliferation, and cell migration [41]. Akt can mediate the activity and/or expression of several pro-and anti-angiogenic regulators [38]. According to previous studies, VEGFR-2 signaling induces Akt activation (p-Akt-1), which in turn activates endothelial NO synthase (eNOS) and regulates the expression of VCAM-1 in the downstream pathway [38, 42]. Angiogenesis needs the embellishment of endothelium-derived NO. Pro-angiogenic factors can promote the production of NO from ECs, which facilitates several processes regulating angiogenesis [43]. In our opinion, it seems that the defective VEFDR-2/Akt axis participated in the dysregulation of downstream proteins and even aberrant angiogenesis. Besides, in line with Sui et al*.* [44], we found that expression of an angiogenic miR-126 was down-regulated, this phenomenon may describe a decrease in Akt and inhibit on other arms of the VEGFR-2 signaling pathway [45, 46], however, further discovery may exactly uncover possible mechanisms involved. MMP-9 is another angiogenic protein that we observed a decrease in the protein level, proposing an inhibition in angiogenesis switch on through the MMP-9/VEGFR-2 pathway in a paracrine manner [47, 48]. Another factor that confirmed an inhibition in angiogenesis is IL-6 and we found a decrease in its protein level [49]. This proinflammatory protein induces angiogenesis through VEGF/VEGR-2 signaling [50]. In addition, we observed that the expression of miR-373 increased. This miR has been reported to play roles in the proliferation and invasion of tumor cells. Ruan et al*.* reported that miR-373 up-regulated angiogenesis in the hypoxic condition [51]. However, the exact function of miR-373 is not fully understood [52]. Based on our knowledge, this is the first report and further studies, which would be taken miR-373 into account, uncover its main function under H_2_O_2_ treatment. We also found that expression of aniogeneic miR-126 was down-regulated in treated cells. This molecule is ECs specific that promotes angiogenesis via suppressing PIK3R2 and endogenous VEGF repressors SPRED1 [45]. Regarding these findings, we, therefore, suggest that the expression pattern of these miRs may correlate to HUVECs impairment and senescence. Our work has limitation, hence the present study has only investigated the effect of H_2_O_2_ treatment on VEGFR-2 downstream signaling; and further investigation such as inhibition and/or stimulation assays are necessary to explain the mechanisms behind VEGFR-2 signaling in this situation.

In summary, through in vitro scratch and LOX activity assays, we found that the migration ability of treated HUVECs was declined. Therefore, we performed the molecular analysis on possible signaling and demonstrated the expression of angiogenic factors, including miR-126, VEGFR-2, HSP-70, VCAM-1, MMP-9, IL-6 as well as Ang-2/Ang-2 ratio and NO production decreased, which suggests an inhibition in angiogenesis. These factors may drive VEGF/VEGFR-2 signaling pathway, which was seemingly inhibited in our experiment.

## Conclusions

We have shown that H_2_O_2_, oxidative stress, decreased angiogenic ability of HUVECs perhaps through VEGFR-2 signaling cascade.. We observed that the expression of angiogenic factors, including miR-126, VEGFR-2, HSP-70, VCAM-1, MMP-9, p-Akt-1, IL-6 as well as Ang-2/Ang-2 ratio and NO production decreased, which suggests an inhibition in angiogenesis. Taken together, our results suggest an evidence of impaired angiogenic signaling, which may be a target for the treatment of age-related diseases. Moreover, our study may provide further insights into VEGFR-2 signaling role under oxidative stress condition. Further scrutiny is needed to uncover the exact underlying mechanisms implicated in impaired angiogenesis in senescent endothelial cells.

## Methods

### Cell culture

Human umbilical vein endothelial cells (HUVECs) purchased from (Pasture, Iran) grown-up in high glucose Dulbecco’s modified Eagle’s medium (Gibco) supplemented with 10% fetal bovine serum (FBS) and 1% penicillin/streptomycin (Gibco). Cells were incubated in a 95% humidified atmosphere of 5% CO_2_ at 37 °C (CO_2_ Incubator; Memert) and every two to three days medium was replaced with a fresh complete medium. For all experiments, cells of passages between 3 and 6 were used.

### Induction of aging

For the induction of aging, HUVECs were seeded into proper cell culture plates with proper density in full growth media and kept for 24 h. Next, cells were exposed to a medium containing hydrogen peroxide (H_2_O_2_, 100 µM) for 24 h. One cell group experience the same conditions without H_2_O_2_—intervention and was kept as control cells. All experiments were completed in triplicate.

### In vitro scratch assay

In vitro scratch assay was done by seeding 5 × 10^5^ HUVECs in 6-well plates to grow to a confluent monolayer. After attachment, monolayers were scratched with a 100 μl sterile pipette tip to make a scratch between the cell monolayer. The scratched monolayer was washed with PBS and then incubated either with basal medium or with H_2_O_2_-containing medium for 24 h and 48 h. images of scratch healing were taken with a phase-contrast microscope (Olympus Corporation) immediately and 24 h and 48 h after treatment at 40 × magnification. Images were then analyzed using ImageJ software (NIH). The HUVECs migration ability was measured by the formula as followed: image area at 24 h or 48 h/initial image area *100.

### LOX assay

The LOX activity assay was used to explore LOX enzyme activity in the cell through a simple calorimetric assay by a commercial kit (KIAZIST, Iran). Briefly, after the treatment period, cells were lysed by LOX Lysis Buffer containing a protease inhibitor cocktail (Cat number: ab271306) and then adopted to freeze-melt-refreeze cycle. The cell lysate was centrifuged at 12,000 rpm at 4° C for 15 min and the supernatant was collected. Protein concentration determined by BSA assay. Then, 50 µl of each sample were mixed with 250 µl LOX Substrate Buffer, 5 µl HRP, and 1 µl Lox Probe and poured into per well of a 96 well plate. Optical density was recorded at points 0, 10, 20, 30, 40, 50 min at 570 nm using a microplate reader (Biotek). LOX activity calculated as Δ570 treatment / Δ570 control.

### NO assay

We calculated NO concentrations according to the enzymatic production of nitrate to nitrite by nitrate reductase in HUVECs by Griess reaction and using a commercial kit (KN096, KIAZIST). Briefly, cells were lysed by NO Buffer supplemented by a protease inhibitor cocktail and then the mixture was exposed to three freeze-melt-refreeze cycles. The cell lysate was centrifuged at 12,000 rpm at 4° C for 15 min and the supernatant was collected. After adding Deproteinizer 1 and 2, samples were centrifuged at 6000 × g for 10 min and supernatant were collected. Then, equal volumes of samples and Griess reagent (a mixture of reagent A, B, and C) were mixed and kept at room temperature for 12 h. the absorbance of each sample was measured at 545 nm by a microplate reader (BioTek). The standard curve was calculated by using serial dilutions of nitrate.

### Real-time quantitative array PCR (qPCR)

Gene expression of VEGR-2, Hsp-70, Angiopoirien-1, and Angiopoietin-2, as well as miR-126 and miR-373, was measured by RNA isolation and subsequent qPCR. After treatment with H_2_O_2_, total RNA in cells was extracted with TRIzol (Invitrogen). The purity and concentration of the RNA were measured with a nanodrop system (Biotek, USA). cDNA of mRNAs and miRNAs were produced using the cDNA Reverse Transcription kits (Cat: A101161, Iran) and (Cat no: BN-0011.17, Iran) according to the manufacturer’s instructions, respectively. Q-PCR was done with the SYBR-green PCR master mix (Cat no: YT2551, Iran) for mRNAs and SYBR Green High ROX Master mix for miRNAs in a MIC Real-Time PCR System (Swiss). Target genes were normalized against the relative reference genes. The RNA transcription levels of genes were measured by the comparative 2 (− ΔΔCT) method. Sequences of primers are prepared in Table 1.

**Table 1:** list of primers

Gene	Primers	Tm (°C)
VEGFR-2	F: CCAGCAAAAGCAGGGAGTCTGT R: TGTCTGTGTCATCGGAGTGATATCC	60
HSP-70	F: GCCGAGCATTCTCTGATCCA R: AACACTTTCGGCTGTCTCCT	60
GAPDH	F: TTGACCTCAACTACATGGTTTACA R: GCTCCTGGAAGATGGTGATG	59
miR-373	5ˊ-GCTACGATTTTGGG-3ˊ	60
miR-126	5ˊ-CAGCGTACCGTGAGTA-3ˊ	60
Snord-47	5ˊ-ATCACTGTA AAACCGTT-3ˊ	60

### Immunoblot analysis

For immunoblot analysis, total protein extracted by RIPA lysis buffer supplemented with protease inhibitor cocktail (Sigma) on ice for 40 min. The protein concentration was reported by a nanodrop system (BioTak). Equivalent amounts of protein (100 mg) were subjected to 10% SDS–polyacrylamide gel electrophoresis. Then, proteins were transferred to the PVDF membrane and incubated with a blocking buffer containing 5% non-fat powdered milk in TBST buffer at room temperature for 1 h. Membranes were incubated with primary antibodies (Cat numbers: sc-393859; sc-28343; sc-47778;sc-293125; ab134047) at 4 °C overnight. After thrice washing with TBST buffer, HRP-conjugated secondary antibody (Cat numbers: sc-516102 and sc-2357) was added to membranes and kept for 1 h at room temperature. Finally, Blots were imagined with a chemiluminescence imaging system (BioRad) and the relative target protein expression was measured using a value ratio of the target band against the protein reference (β-actin) by Image J software version 1.52a.

### Statistical analyses

T-test was performed using GraphPad Prism version 8.0.1 (California USA) to evaluate the differences between the groups. All experiments were replicated in three sets and results were presented as mean ± SD. A *p*-value of 0.05 was considered statistically significant.

## Supplementary Information

**Additional file 1.** Supplementary information.

## Data Availability

Data and materials are available from a request to corresponding author.

## Abbreviations

ECs: Endothelial cells
H_2_O_2_: Hydrogen peroxide
CVD: Cardiovascular diseases
eNOS: Endothelial NO synthase
HUVECs: Human umbilical vein endothelial cells
