# Supplementary figures and images for: Angiogenic ability of human endothelial cells was decreased following senescence induction with hydrogen peroxide: possible role of vegfr-2/akt-1 signaling pathway
Source: BMC Mol Cell Biol. 2022 Jul 25;23:31. doi: 10.1186/s12860-022-00435-4 (PMC9310472; doi:10.1186/s12860-022-00435-4)

**Supplementary Information
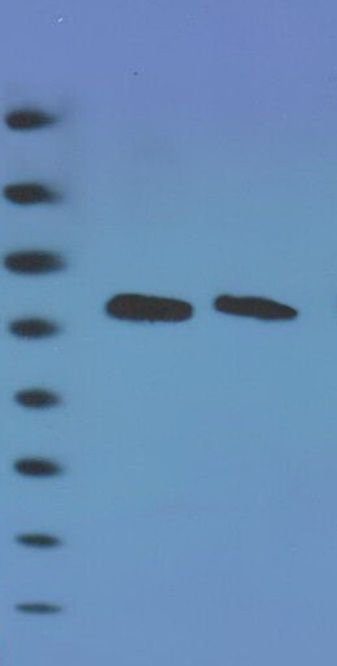
**

**P-AKT-1**

**
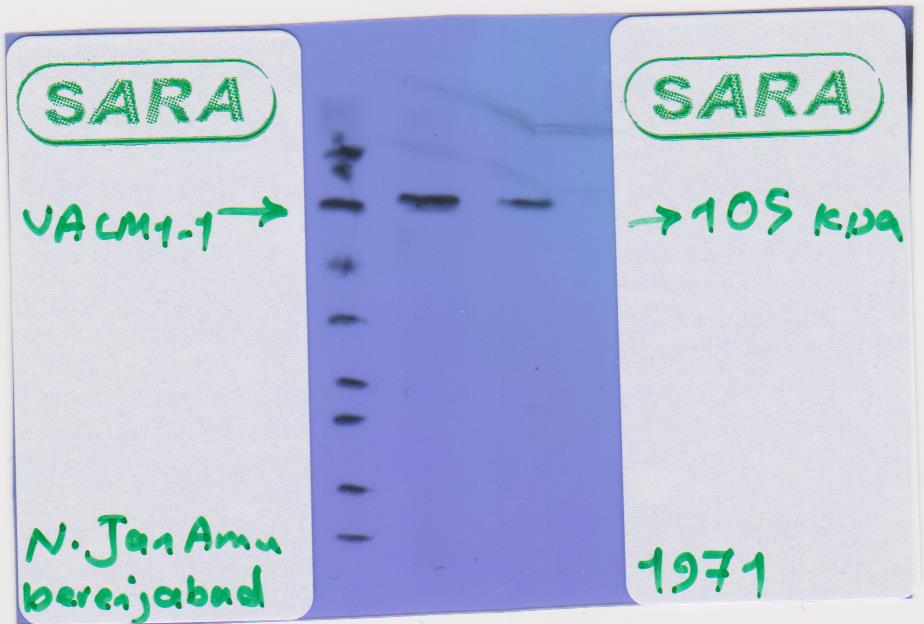
**

**VCAM-1**

**
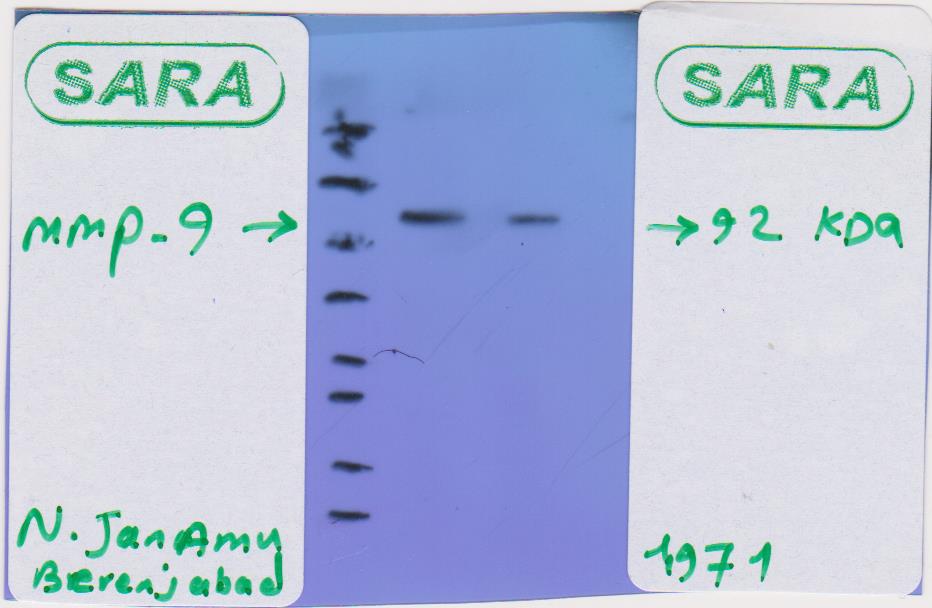
**

**MMP-9**

**
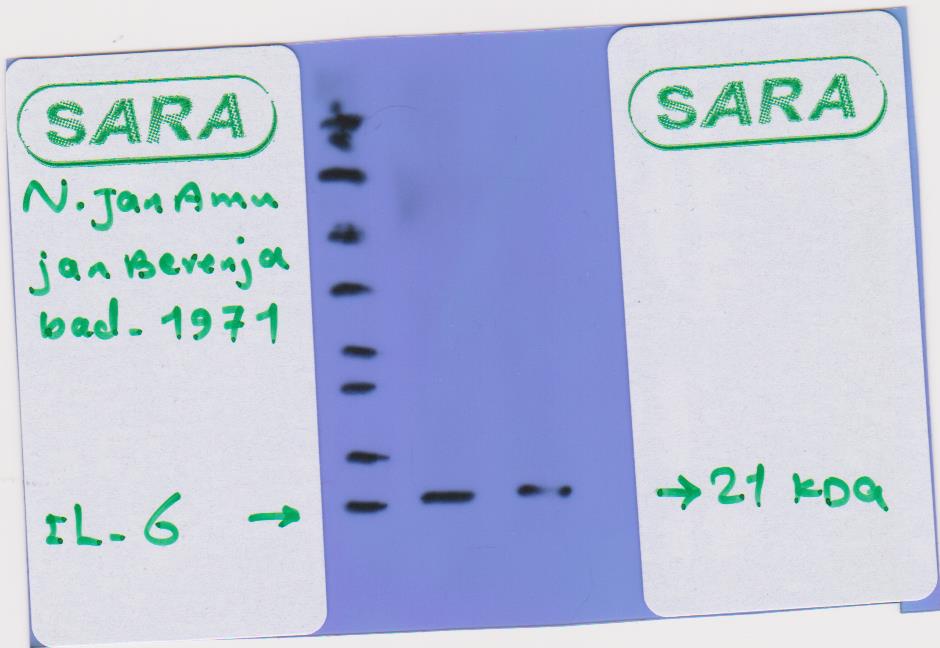
**

**IL-6**

**
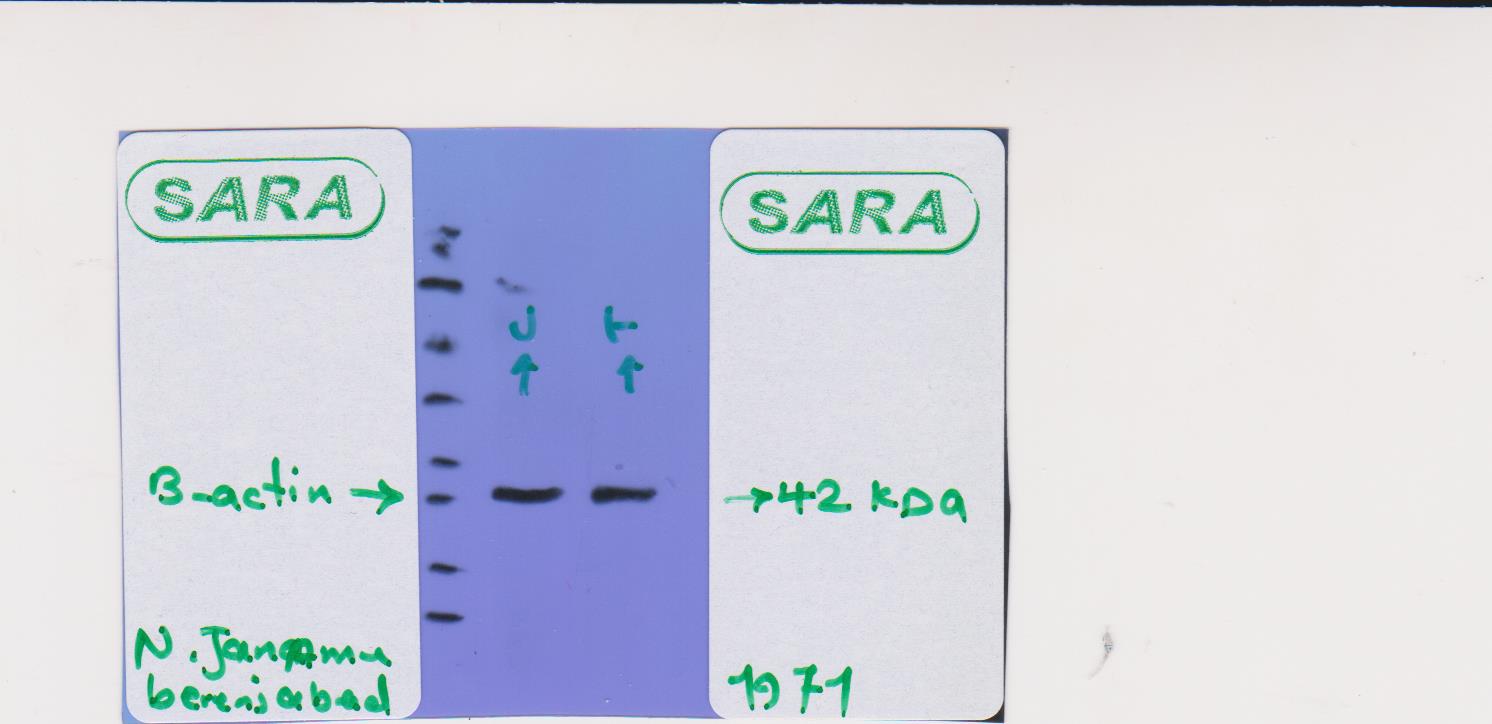
**

**B-actin**

Supplement: Supplementary file 1 — Additional file 1. Supplementary information. [file 12860_2022_435_MOESM1_ESM.docx]
